# Oral State and Salivary Cortisol in Multiple Sclerosis Patients

**DOI:** 10.3390/biomedicines12102277

**Published:** 2024-10-08

**Authors:** Aleksandra Kapel-Reguła, Justyna Chojdak-Łukasiewicz, Anna Rybińska, Irena Duś-Ilnicka, Małgorzata Radwan-Oczko

**Affiliations:** 1 Private Dental Practice AL-DENTA, 48-303 Nysa, Poland; 2 Department of Neurology, Faculty of Medicine, Wroclaw Medical University, 55-556 Wroclaw, Poland; justyna.chojdak-lukasiewicz@umw.edu.pl; 3 Department of Oral Pathology, Faculty of Dentistry, Wroclaw Medical University, 50-425 Wroclaw, Poland; anna.szczygielska@umw.edu.pl (A.R.); irena.dus-ilnicka@umw.edu.pl (I.D.-I.); malgorzata.radwan-oczko@umw.edu.pl (M.R.-O.)

**Keywords:** multiple sclerosis, salivary cortisol, oral state, dental care

## Abstract

**Background:** MS patients experience gradual and progressive functional limitation, bulbar symptoms, cognitive dysfunction, and psychiatric disorders that can impinge on oral status. This study aimed to investigate the oral state, oral hygiene habits, and salivary cortisol levels in patients with relapsing-remitting multiple sclerosis (RRMS) compared to healthy controls. It also evaluated systemic parameters: disease duration, type of Disease Modifying Therapy (DMT), disability score, professional activity, and smoking in the study group. **Methods:** This study included 101 patients (71 women and 30 men, aged 16–71 years) and 51 healthy volunteers (36 women and 15 men, aged 28–82 years). The oral examination assessed the number of teeth, type and number of dental fillings and prosthetic restoration, oral hygiene state, and salivary cortisol. **Results:** It was found that MS patients had significantly more professional activity, swallowing problems, pronunciation issues, dry mouth, and taste disturbances than the control group. They brushed their teeth twice daily significantly less often. The API was higher, while the SBI was lower in MS patients. Disease duration positively correlated with age and number of missing teeth. The Expanded Disability Status Scale positively correlated with age, disease duration, number of missing teeth, number of composite fillings, and right and left-hand Nine Hole Peg test scores, and negatively correlated with the Sulcus Bleeding Index. Salivary cortisol levels did not differ between groups and correlated only with the disability scale. **Conclusions:** MS patients require ongoing dental care and preventive measures to manage both general and oral health symptoms effectively.

## 1. Introduction

Multiple sclerosis (MS) is an autoimmune, chronic, inflammatory, demyelinating, and neurodegenerative disease of the central nervous system (CNS) that damages myelin sheaths, oligodendrocytes, nerve cells, and axons [1,2,3,4]. Epidemiological studies indicate that the condition mainly affects people between the ages of 20 and 40, although MS can develop in childhood or after the age of 60 [3,5,6,7]. Women are affected twice as often as men [8].

The disease is characterized by a gradual and progressive reduction in functional and cognitive abilities, eventually leading to disability [1]. Patients with numbness in the arms and hands may have difficulty holding objects, such as toothbrushes. Tremors can affect the head, jaw, mouth, and speech, causing loss of coordination. Motor disabilities can make it difficult to access doctors’ offices, including dental surgeries. Depression, often accompanying MS, reduces the motivation to take care of personal hygiene, including oral hygiene. Another aspect of MS is the common use of corticosteroids, immunomodulators, immunosuppressants, and other pharmaceuticals, which can affect the oral mucosa, increasing the risk of caries and periodontal disease [9,10]. Additionally, the temporomandibular joint dysfunction that occurs in some patients [11] may contribute to chronic pain [12].

The etiology of MS is unknown. It is widely believed that infectious, genetic, immunological, and environmental factors are involved [13]. It is noteworthy that studies confirm a positive association between a stressful experience and the risk of exacerbation of MS symptoms [14,15]. Scientific publications have highlighted the positive association between a stressful experience and the risk of exacerbation of MS symptoms. Cortisol plays a key role in the stress response [16]. Mohr et al. [15] showed that reducing stress in people with MS could reduce the production of interferon gamma, the substance responsible for disease relapse and progression. In another study, Sapolsky et al. [17] investigated that a small increase in cortisol levels, observed during high-stress events, increased the sensitivity of T cells to a number of cytokines and peptides that promote a pro-inflammatory response.

Some studies have shown that the hypothalamic–pituitary–adrenal (HPA) system is overactive in patients with MS. In many analyses, cortisol levels were found to be elevated in MS patients, regardless of how they were assessed (blood, urine, saliva) [18]. Saliva, however, offers readily available samples [19], as it can be obtained non-invasively, repeatedly, without any risk of infection, and in a cost-effective manner [20].

The purpose of this study was to evaluate oral status and oral health and hygiene in relation to systemic parameters and salivary cortisol levels in patients with relapsing-remitting multiple sclerosis (RRMS) compared to a control group of healthy volunteers.

## 2. Materials and Methods

### 2.1. Study Participants

The patients of the study group were recruited by the Department of Neurology, Wroclaw Medical University, from March to November 2023. The diagnosis for MS was made according to McDonald’s criteria [21]. All patients were under disease-modifying therapy (DMT) and had regular follow-up visits. Finally, 101 MS patients were included in the study group. In the control group, 51 generally healthy individuals were recruited. At the end of this examination, each patient was informed about his/her oral cavity status and required treatment and/or improvement of oral hygiene. 

### 2.2. Ethical Considerations

This study was approved by the Bioethics Committee of Wrocław Medical University, Poland (approval no. KB-14/2023) and conducted in accordance with the Helsinki Declaration and Good Clinical Practice. All patients were informed about this study’s objective, and before the clinical assessment, every patient signed an informed consent form to undergo the oral examination.

### 2.3. Oral Status

A single calibrated dentist performed a complete oral cavity examination, assessing the number of natural teeth, the type and number of dental fillings, the presence and type of prosthetic restorations, and the state of the oral mucosa. Oral status was assessed according to the Approximal Plaque Index (API) and Sulcus Bleeding Index (SBI) [22]. During the same assessment, plaque remnants on the probe and bleeding on contact with the gingival sulcus were noted, with a maximum of 28 points. The number of positive points for each of these two indices was divided by the number of measurements to yield a percent plaque and bleeding index. In the further general anamnesis, the local oral and behavioral factors and oral problems were gathered. 

### 2.4. Medical Records

The demographic and clinical data were retrieved from the patient’s medical record with compressive documentation of the treatment and progression of the MS. Duration of disease, degree of disability—expressed as Expanded Disability Status Scale (EDSS)—type of DMT, and type of MS were determined. EDSS scores are based on eight functional systems (FS) and walking. The scale ranges from 0 (normal) to 10 (death from multiple sclerosis) [23]. In an assessment of the dysfunctions of the upper limbs, the 9-Hole Peg Test (9-HPT) was used [24,25]. This test was administered by asking the subject to take small pegs from a container, one by one, and place them into holes on a board as quickly as possible, then remove the pegs from the holes, one by one, and replace them back into the container. Scoring is recorded in seconds and is based on the mean time taken to complete the test using each hand separately. The score, in seconds, for each arm was recorded twice, and the mean of the two trials was calculated. Subsequently, the 9-HPT score resulted from the value obtained and subtracting the worst and the best scores of the two 9-HPT scores from both the left and right hands [24,25]. 

### 2.5. Saliva Samples

Saliva samples were taken from 101 patients (experimental group) and from 51 healthy people (control group). After 5 min of rest following clinical examination, participants were asked to spit out approximately 2–5 mL of unstimulated saliva into a 5 mL sterile Eppendorf-like tube in a sitting position and slightly leaning forward. More precisely, the saliva sample was provided by spitting when enough of the biosample had been stored in the bottom of the mouth without forcing the spitting. The collection time varied from 5 to 20 min. Each study participant was assigned an identification number, which was used to sign the test tubes containing saliva to ensure the pseudo-anonymization of the process. The collected saliva samples were centrifuged at 10,000× *g* for 10 min at 4 °C. Obtained supernatants were aliquoted into three sterile 1.5 mL tubes and conserved at −80 °C until use.

#### Cortisol Level Diagnostics in Saliva

For the quantitative determination of cortisol in saliva samples, we used a commercially available kit, the Cortisol ELISA Kit (DRG International, Springfield, NJ, USA), which is based on a competitive enzyme-linked immunosorbent assay (ELISA). In this competitive binding technique, the microtiter wells were coated with a monoclonal antibody directed toward an antigenic site on the cortisol molecule. 

More precisely, after pipetting all reference samples from the standard curve and saliva samples from study participants into microtiter wells, the endogenous cortisol present in the sample competed with the subsequently added horseradish peroxidase (HRP)-labeled cortisol (enzymatic conjugate) for a limited number of binding sites on a monoclonal antibody previously coated in the wells. 

After removing previously unbound material (HRP-labeled cortisol), the substrate solution (TMB—tetramethylbenzidine) was added to all wells to develop the color. The color development was then stopped (using stop solution—0.5 M H_2_SO_4_), and the absorbance values were measured spectrophotometrically at 450 nm using an Epoch Microplate Spectrophotometer (US BIOTEK LABORATORIES, Shoreline, WA, USA). The intensity of the color was inversely proportional to the concentration of endogenous cortisol in samples. A standard curve was generated by plotting the absorbance values for each used standard (0 ng/mL, 20 ng/mL, 50 ng/mL, 100 ng/mL, 200 ng/mL, 400 ng/mL, and 800 ng/mL) on a linear y-axis against its concentration on a logarithmic x-axis. On the basis of the standard curve, the concentration of cortisol in saliva samples from study participants was calculated. The range of the assay was 1.3–800 ng/mL, with an analytical sensitivity of 1.3 ng/mL. The unit conversion factor was 1 ng/mL = 2.76 nmol/L.

### 2.6. Statistical Analysis

A statistical analysis was performed using the EPIINFO Ver. 7.2.3.1 and Statistica Ver. 13.3 software packages. The results of this study were subjected to statistical processing. For study groups, the number of cases (N), mean, standard deviation (SD), median, range (min–max), and lower and upper quartile (25Q–75Q) of the quantitative parameters were calculated. Depending on the distribution, quantitative data were presented: 1. as mean ± SD, in the case of variables with normal distribution; and 2. as median and interquartile range M (25Q ÷ 75Q), in the case of variables with non-normal distribution. Qualitative variables were presented as absolute values and percentages (%). The normality of distribution was checked using the Shapiro–Wilk test, and the homogeneity of variance was checked using Levene’s test. The statistical significance between means for different groups was calculated by a one-way analysis of variance (ANOVA), alternatively using the non-parametrical U Mann–Whitney test (for two groups) or Kruskal–Wallis test (for three or more groups) when the variances in groups were not homogeneous. The relation between the two parameters was assessed using correlation analysis, and Spearman correlation coefficients (R) were calculated. Statistical significance between frequencies was calculated by the chi-square test χ^2^_df_ with a corresponding degree of freedom of [df = (m − 1)*(n − 1), where m—number of rows, n—number of columns]. To reject the null hypothesis, a *p*-value of less than 0.05 was required. 

## 3. Results

The characteristics of the study groups are shown in Table 1. The study group of 101 MS patients included 71 women and 30 men aged 16–71, with a median age of 44.0. All patients had a diagnosis of RRMS and were treated with four types of DMT: interferon in 16 patients, immunomodulating factors in 56, immunosuppressive agents in 16 patients, and monoclonal antibodies in 13 patients. As much as 53.5% of patients in the RRMS group had coexisting general diseases. In the control group, there were 51 age- and sex-matched healthy volunteers, 36 women and 15 men, aged 28–82 years, with a median age of 44.0 years. In comparison with the control group patients, the RRMS study group had a significantly higher number of missing teeth (4.57 vs. 1.65, *p* = 0.02), a higher number of amalgam fillings (0.82 vs. 0.49, *p* = 0.03), and the API% was also significantly higher (41.5% vs. 27.4%). On the other hand, the number of composite fillings (9.03 vs. 11.6, *p* = 0.00) and the SBI% indices (26.9% vs. 47.5%) were significantly lower in this group when compared to the control group. The salivary cortisol concentration was similar in both groups. There was also no difference between the groups in the number of teeth for treatment or extraction.

**Figure 1:**
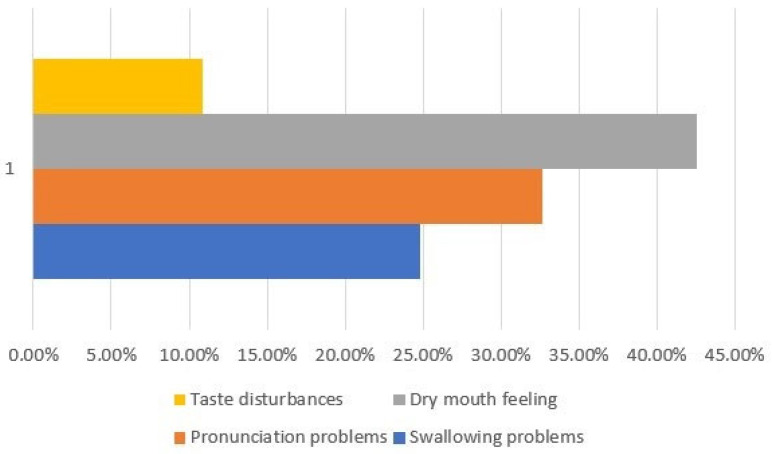
Description of most of the significantly related parameters in patients with MS.

However, there were notable differences, with *p* ≤ 0.05 in parameters between the RRMS group and the control group gained from the anamnesis and presented in Table 2. In the study group, there were significantly fewer subjects with professional activity (60.4% vs. 88.2%). RRMS patients had significantly more frequently present symptoms such as swallowing problems (24.8% vs. 1.96%), pronunciation difficulty (32.7% vs. 1.96%), dry mouth feeling (42.6% vs. 15.7%), and taste disturbances (10.9% vs. 1.96%), but the twice-daily tooth-brushing frequency was lower in this group (79.0% vs. 92.1%), as presented in Figure 1.

The differences between the investigated groups in the next parameters were only borderline statistically significant. In the RRMS group, metallic taste feelings were more frequently present (12.8% vs. 3.92%), and the presence of gingival problems was more frequently indicated (25.7% vs. 13.7%). In self-assessment, bad oral hygiene was more frequently indicated (8.0% vs. 3.9%). These patients also visited dental offices less frequently last year (32.7% vs. 43.1%). There was one edentulous patient in the study group (54-year-old woman) with full removable prostheses. In both groups, there was a similar percentage of smoking patients—12.9% in RRMS and 11.8% in the control group. During the clinical examination, no pathological lesions were present on oral mucosa, both in the RRMS and healthy groups. 

Table 3 presents the correlations between the investigated parameters. The duration of the MS disease was correlated positively with patients’ age (r = 0.47; *p* = 0.00), the number of missing teeth (r = 0.27; *p* = 0.00), the level of the EDSS (r = 0.53; *p* = 0.00), and the 9-HPT times for the right (r = 0.45; *p* = 0.00) and left (r = 0.48; *p* = 0.00) hands describing the upper hand disfunctions. The EDSS score was positively correlated with patient age (r = 0.58; *p* = 0.00), the number of missing teeth (r = 0.44; *p* = 0.00), the 9-HPT times both for the right (r = 0.71; *p* = 0.00) and left (r = 0.66; *p* = 0.00) hands, and it was negatively correlated with the SBI% (r = −0.23; *p* = 0.02). The correlation between the EDSS and the number of composite fillings bordered statistical significance (r = 0.19; *p* = 0.06). No correlation was observed between salivary cortisol concentration and the investigated parameters. In the control group of healthy people, there were no significant correlations present between the evaluated parameters. 

Further observations revealed a higher level of salivary cortisol (on the border of statistical significance [p** = 0.07]) in patients treated with interferon and immunosuppressive agents. The RRMS time duration was significantly longer in patients without professional activity (p* = 0.02), with swallowing problems (p*** = 0.00), dry mouth syndrome (p* = 0.02), pronunciation problems (p* = 0.00), and in patients using a manual toothbrush (p** = 0.02). The disease duration was also longer in the group of patients treated with interferon (p** = 0.055), but only near the border of significance.

However, the EDSS level scale was significantly higher in patients without professional activity (p** = 0.00), in patients with swallowing problems (p*** = 0.00), in patients with pronunciation problems (p* = 0.00), and in patients with dry mouth feelings (p*** = 0.00). Also, patients who needed help during tooth brushing (p*** = 0.00), those using manual toothbrushes (p** = 0.01), those using a movable prosthesis (p** = 0.00), and those treated with monoclonal antibodies (p** = 0.00) showed significantly higher scores on the EDSS. Additionally, on the border of statistical significance (p** = 0.08) was the result of the EDSS being higher in patients with higher education. Statistical tests used: ANOVA*, Kruskal–Wallis Test**, Mann–Whitney U Test***.

## 4. Discussion

The aim of the present study was to assess the oral condition of MS patients in comparison to healthy people and to describe the connection of oral parameters with some of the general MS parameters. The control group was matched according to the age and gender of patients. 

Studies around the world show that women are twice as likely as men to develop MS [26]. In our investigation of 102 patients, women made up as much as 70% of the study group. This result is comparable to data obtained in other publications [3,27,28]. The positive discovery is that the patients’ salivary cortisol levels are normal, indicating that despite their difficult health journey, they maintain acceptable stress levels, at least to the extent that their oral health is not adversely affected.

MS patients experience gradual and progressive functional limitation, bulbar symptoms, cognitive dysfunction, and psychiatric disorders [7] that can impinge on oral status. Therefore, in this study, we compared the oral condition of people with MS and healthy volunteers. In the MS group, we found a higher number of missing teeth, 4.57 vs. 1.65, respectively. Similar observations were noted in a study of 21 Brazilian patients (6.86 vs. 3.74) [29], in 50 Croatian patients (7.04 ± 3.89 vs. 3.94 ± 2.74) [30], and during an investigation conducted in Iran, in 50 patients (1.12 vs. 0.20) [31], the number of teeth lost in the study group was lower. In a Turkish study of 92 MS patients [32], both in the investigated group and in healthy volunteers, only two teeth, on average, were missing. In contrast, in an Australian group of 56 patients [33], the authors found a lower number of missing teeth (5.4) when compared to the healthy group (6.2). The reason for the observed differences in the number of lost teeth may be due to motor disabilities making daily tooth brushing difficult, depressive symptoms resulting in a lack of desire for oral hygiene, architectural barriers preventing access to dental offices, and side effects of the therapies used.

Several studies [34,35,36,37] showed that exposure to low doses of metals, including mercury, can cause some autoimmune diseases, such as MS, rheumatoid arthritis, and systemic lupus erythematosus. In our study, MS patients had a significantly higher number of amalgam fillings and fewer composite fillings compared to the control group. The topic of amalgam fillings was also addressed by a Romanian study [28], where 90% of patients with amalgam fillings had gingivitis. In our study, fewer fillings were found in MS patients overall than in healthy controls (9.85 vs. 12.06). Kovac et al. [30] described similar results (4.30 ± 4.69 vs. 7.24 ± 5.36) and (0.96 vs. 1.80) [31]. The exceptions were an Australian study [33], where the average number of filled teeth in both groups was similar (3.6 vs. 3.15). It can be speculated that the reason for this condition is the postponement of dental visits for emotional and motor reasons by patients with MS, which prevents conservative treatment of the teeth, making extraction necessary. A similar conclusion was reached by Mortzavi et al. [31], claiming that MS patients mostly preferred to have their teeth with the caries removed rather than restored.

In this study, the mean DMF in the patients and control group was similar, 15.33 and 14.25, respectively. The results from a Croatian study [30] with a DMF of 12.58 vs. 11.72 and from the Australian study [33] with an average of 10.6 vs. 10.3, showed marginally better teeth conditions with the lower DMF. On the other hand, in an Iranian population [31], the level of the DMF index in both groups was similar and at a very low level (2.64 vs. 2.16). Meanwhile, in a Turkish investigation [32], the DMF index in patients with MS 5.4 was lower than in the control group 6.2. 

Despite the comparable age range of our group (16–71 years), an Italian (12–70 years) study investigated patients [38], and the removable prosthetic restorations were used by only 8.91% of Polish MS patients compared to 26% of Italian patients. However, the Italian study [38] was based only on a questionnaire, without information about the number of missing teeth. The lower number of prosthetic restorations in Polish patients may be due to the greater number of teeth they had, the presence of the fixed prosthetic restorations, their reluctance to use any prosthetic restorations, or the lack of funds financed to make them.

Similar to periodontal diseases, MS has an inflammatory basis, so a link between the two conditions is likely [9]. Also, it is noteworthy that medications taken to restrain the progression of the disease and relieve symptoms can cause side effects [9]. In our study, it was observed that despite significantly higher plaque accumulation (API 41.5% vs. 27.4%), the bleeding index (SBI) was significantly lower (26.9% vs. 47.5%) in the study group in comparison to healthy patients. However, the signs of gingival bleeding during tooth brushing were reported in a similar percentage of patients, 22.7% vs. 23.5%, when comparing the MS and control groups. In a Turkish study [32], lower SBI values in RRMS patients, 18.08 vs. 20.19 in the healthy group, were also reported. Spanish researchers reported similar and very important observations [39] that although 65% of the studied patients had calculus deposits, only 5% of them demonstrated gingival bleeding. We presume that the effect of this connection is due to MS medications. Currently, each of the DMTs is used to target other components of the innate or acquired immune system, aiming for a broadly anti-inflammatory effect [40]. The mechanism of action is based on a reduction in the number of B lymphocytes, blocking lymphocytic infiltration, reducing antigen presentation to B lymphocytes, and modulating pro-inflammatory cytokine secretion by lymphocytes [41]. The aforementioned mechanisms are simultaneously factors that can reduce gingival inflammation manifested by gingival bleeding despite the presence of the main cause, i.e., the accumulation of plaque and calculus deposits [42].

The rationale behind the disease’s symptoms, namely weakness, ataxia, and spasticity [7,40] may limit the ability to effectively perform daily oral hygiene on a suitable level. There is also a non-negligible emotional aspect associated with frequent depressive episodes of MS patients [2,43] and the resulting lack of desire to take care of hygiene. The results obtained in our study do not seem to support this thesis. An essential conclusion from this presented study is that patients with MS perform a general visit to the dentist regularly and maintain good oral hygiene. This performance demonstrates a commitment between leading dentists and neurologists, as well as patients. Our study notes that 79% of patients reported brushing their teeth twice a day, in comparison to 92% of healthy volunteers, and only 8.0% of them self-assessed their oral hygiene as bad. Moreover, only 3.9% of the study group reported a need for help during tooth brushing. In another Polish study [27], 61.8% of patients brushed their teeth twice a day, while 17.1% brushed them three times daily or more. Covello et al. [38] noted that 58% of Italian patients also performed oral hygiene procedures twice a day. In a Turkish study [32], brushing teeth twice a day was reported by a similar percentage of patients and healthy volunteers (47.6% vs. 42.4%).

Another very important aspect of oral hygiene is the utensils used. Many studies [44,45,45,46] confirm that electric toothbrushes are more effective in removing up to 11%–21% more dental plaque than manual toothbrushes. The undeniable advantage of these devices is their simple operation, the lack of need for precise hand movements, and the ease of manipulation of the small brush head, which is especially important in people with physical dysfunctions and limitations in the stomatognathic system with mouth opening difficulties. These factors reduce brushing time and facilitate hygiene procedures in people with limited motor skills or their caregivers. Despite the undoubted advantages of electric toothbrushes, a larger group of patients, 64.4% in our study, used manual toothbrushes (vs. 35.6%). A similar result was reported in an Australian study [33], where 67.9% of patients used a manual toothbrush. However, in an Italian study [38], a reverse ratio was observed (13% vs. 71%, with the majority of patients using electric toothbrushes). The reason for the more frequent use of manual brushes may be a lack of awareness or a well-established tradition, as electric toothbrushes only appeared in the 1960s and came into widespread use in the 1990s [47]. It is also not insignificant that an electric toothbrush is evidently more expensive than an electric one, and because of that difference, it is very often less available.

MS is a progressive disease, and its associated symptoms, such as ataxia, tremors, loss of coordination, slurred speech, fatigue, depression, and progressive disability, can affect motivation to visit the dentist’s office and significantly hinder dental treatment [10]. According to our study, 44.6% of Polish patients and 49% of healthy volunteers declared that they visit the dentist every six months, while 32.7% of patients and 43.1% of individuals in the control group visit the dentist every 12 months. In an Italian study [38], 71.4% of patients with MS visited a dentist at least once a year. In Australia [33], patients with MS were more likely to visit the dentist’s office than healthy people, and the highest percentage of them visited more often than every six months (44.6% vs. 41.8%).

Thus, patient awareness, relatively low disability, low average age, and illness duration seem to play key roles in oral hygiene care. 

Among other symptoms, the sensation of dry mouth, chronic autoimmune diseases, and depression can be a side effect of certain medications. Xerostomia is a symptom of systemic diseases leading to impaired salivary gland function [48]. According to the literature, more than 500 chemical compounds cause or exacerbate feelings of mouth dryness, including various pharmaceutical groups [49,50]. In a paper by Cockburn et al. [51] that evaluated side effects of MS medications, xerostomia was mentioned most often as an adverse effect.

In our study, 42.6% of MS patients complained of dry mouth feeling. In the Italian patients [38], this problem presented in 57% of subjects. In the Manchery study [33], xerostomia was reported by 64% of patients and was the most commonly reported symptom. A similar observation was described in another study of the Polish population [27]; however, in this scientific description, xerostomia was assessed in patients with the type of secondary progressive MS with a history of steroid use. Our study included patients with the relapsing-remitting form of MS. It is also noteworthy that our patients reporting xerostomia had concomitant diseases and were also taking other medications in parallel to their MS treatment. Thus, it can be presumed that the reported sensation of dry mouth was induced by MS medications, comorbidities, and pharmacotherapy for these conditions. Treatment of xerostomia should be a priority for people with MS. Medications that cause decreased saliva secretion increase the risk of oral diseases such as caries, periodontal diseases, and oral fungal overgrowth [52]. Hence, the standard of care for MS patients with dry mouth should be recommending the use of toothpaste with high fluoride content at home to address the increased risk of tooth decay and the use of gels, oils, and rinses that are saliva substitutes to improve oral mucosa condition and relieve symptoms.

According to Cockburn et al. [51], the most frequently cited side effects of medications used to prevent the progression of MS were sinusitis and taste disorders. In addition, a study on taste disorders in MS [53] showed that a significant number of patients had taste deficits, which were linked to brain damage resulting from MS. In our study, dysgeusia was reported by 10.9% of MS patients. An evidently higher 34% of taste disorders were observed among Italian patients [38]. The discrepancies may be due to the duration of the disease, the type of therapy used, factors related to the progression of the disease, and the number of demyelinating changes in the brain. Additionally, our patients were subjected to four different types of the treatments for MS: interferons, immunomodulating drugs, immunosuppressants, and monoclonal antibodies, as presented in Table 4. These types of treatment comply with the standard procedure of potential treatment available to patients with this disease.

On the basis of strong scientific evidence, Degelman and Herman [54] showed that smoking could influence MS, and the causal relationship between smoking and disease progression was assessed as moderate. Cigarette smoking is known to be a lung irritant that triggers a pro-inflammatory cascade, leading to chronic inflammation. Long-term inflammation can increase the risk of developing autoimmune diseases such as MS due to the cross-reactivity between lung antigens and myelin antigens [55,56]. In our study, the percentage of smokers in both the investigated, 12.9%, and the control group, 11.8%, was similar. Also, Varol et al. [32] presented a similar ratio in the results between MS patients and a control group, 25% vs. 21.75%. However, in an Australian study [33], there were more smokers among patients, 17% vs. 9%, than controls. It is therefore important to make patients, especially those suffering from autoimmune diseases, aware of the harm of smoking and even to implement strategies to combat nicotine addiction.

In our study, there was no difference in salivary cortisol levels between MS patients and healthy volunteers. There was also no connection observed between the cortisol concentration and investigated clinical parameters, except for the positive correlation with disease duration that lacked statistical significance. We determined cortisol levels once in the saliva taken in the morning in both investigated groups. In other studies [43,57,58,59,60], measurements were taken several times a day.

The authors Gold et al. [58] and Kern et al. [43] showed that daily cortisol profiles differed between MS patients with depressive symptoms and healthy volunteers, but there were no differences between non-depressed patients and controls. Similar findings were obtained by Powell [57], noting that differences in cortisol levels between patients and controls appeared after accounting for symptoms of depression and chronic stress. In a study by Hildebrandt et al. [60], analyzing cognitive fatigue in MS patients in relation to cortisol, it was noted that patients with MS but with no cognitive fatigue differed in terms of cortisol levels from healthy subjects. No significant differences were noted for patients with fatigue. Thompson et al. [59] found that cortisol levels helped distinguish healthy people from MS patients, but only those who do not yawn. 

It seems significant that statistically important differences in cortisol levels between patients and healthy controls were found based on yet another factor. Thus, it can be assumed that levels of cortisol fluctuations in patients are due to a component arising from the nature of MS and other psychosomatic factors. The issue requires further research and analysis. 

The duration of MS was positively correlated with patient age, number of missing teeth, the disability scale, and 9-HPT—for both hands. In our examination, the mean EDSS value was 2.49. Similar levels of disability were presented in an Australian study with a value of 2.8 [33], in a Turkish study with a value of 2.81 [32], and in a Romanian study with a value of 2.5 [28]. In our study, the EDSS level was significantly higher in inactive patients with swallowing, speech, and xerostomia problems in older patients, in patients with longer-lasting disease, in patients with a higher number of missing teeth, with a higher number of composite fillings, and with 9-HPT scores of the right and left hands.

The 9-HPT is the gold standard for assessing dysfunctions of the upper limbs (UL) in MS, calculating a mean score from the right and left arms.

In our study, the disability scale scores positively correlated with the dysfunction of both the right (r = 0.71; *p* = 0.00) and left (r = 0.66; *p* = 0.00) hands. In the Solaro et al. study [61], the findings suggested that there was an interaction between mean right–left 9-HPT scores and asymmetry in predicting the disability score (EDSS).

The strength of our study is the quite numerous MS patient groups and the clinical investigation of numerous clinical oral parameters. Observations from our study are comparable to the available results from other investigations. Unfortunately, there are not many such scientific descriptions taking into consideration similar investigated parameters. Some of the papers cited in this work are not current; some describe a rather small size for the investigated MS patient groups, and one is based on a questionnaire—without clinical oral investigation. Because of that, in the discussion, it was difficult to compare a large number of the evident outcomes from the other studies. A limitation of this study is that the cortisol level measurements were only taken from one saliva sample in the morning from each patient before the clinical examination. The level of cortisol could have been different if the saliva had been taken several times during the day. 

**Table 4:** Types of the MS therapies used by the patients in this study.

Numbers of Patients	Type of the Therapy Used	Commercial Names of the Drugs Used	Action of the Drug on the MS	References
15	Interferons	Rebif, Avonex, Betaferon, Plegridy	Not completely known; in effect stabilize dysregulated central nervous system’s inflammation	[62]
56	Immunomodulating drugs	Tecfidera, Copaxone, Gilenya	It affects cells of the innate immune system (monocytes, dendritic cells and B cells) in turn modulating the adaptive functions of B and T cells by stimulating the secretion of anti-inflammatory and regulatory cytokines.	[63]
16	Immunosupressants	Aubagio, Zeposia, Mavenclad	1. Selectively and reversibly inhibits the activity of dihydroorotate dehydrogenase (DHO-DH), which catalyzes the process of pyrimidine synthesis 2. Inhibition of lymphocyte migration to the central nervous system 3. A purine antagonist, a synthetic derivative of deoxyadenosine (dA). Selectively cytotoxic to cells with a relatively high ratio of deoxycytidine kinase to deoxynucleotidase	[64]
13	Monoclonal antibodies	Ocrevus, Tysabri, Kesimpta	Highly precise by targeting molecules displayed on cells involved in distinct immune mechanisms of MS pathophysiology.	[65]

## 5. Conclusions

MS is a chronic disease with a chronic course, and because of the different factors involved in its etiopathology, which are still not fully recognized, it is laborious in its treatment. MS develops in young adults. On the one hand, the disease can influence the oral condition, and on the other hand, different oral conditions can influence the course of MS. Our outcomes confirm these two-way connections. MS patients display worse oral hygiene with higher plaque accumulation but with a lower inflammation level observed as the gum bleeding, that is connected with the anti-inflammatory drugs used in MS course. They have higher number of missing teeth correlated with the disability score and teeth requiring treatment or extraction. They also indicate problems with dry mouth feeling and taste disturbance. The salivary cortisol level was the same in MS patients and the healthy group, which indicates that the disease course does not influence patients’ life too negatively. MS patients require constant oral care and prevention. Examined MS patients had similar behaviors to healthy individuals regarding the frequency of the tooth brushing and visiting the dental office. Only well-educated patients with hygiene instructions repeated during each control visit [66] and proper cooperation in therapy can help to restrain the progression of both the general and oral symptoms of MS. 

## Figures and Tables

**Table 1:** The characteristics and investigated parameters in the RRMS patients and control group.

Variable	RRMS Group	Control Group	Mann–Whitney Test
Number of subjects	101	51	N/A
Gender female/male	71/30	36/15	NS
Age (years)			
mean ± SD	45.7 ± 10.4	44.7 ± 8.2	
median (Q1–Q3)	44.0 (39.0–53.0)	44.0 (40.0–49.0)	NS
Disease duration (years)			
mean ± SD	11.4 ± 7.1	N/A	N/A
median (Q1–Q3)	11.0 (6.0–15.0)		
Number of missing teeth			*p* = 0.02
mean ± SD	4.57 ± 6.74	1.65 ± 1.47	
median (Q1–Q3)	2.0 (0.0–6.0)	1.0 (0.0–3.0)	
Number of composite fillings			*p* = 0.00
mean ± SD	9.03 ± 5.24	11.6 ± 4.1	
median (Q1–Q3)	9.0 (4.0–13.0)	12.0 (9.0–14.0)	
Number of amalgam fillings			*p* = 0.03
mean ± SD	0.82 ± 1.88	0.49 ± 1.70	
median (Q1–Q3)	0.00 (0.0–1.0)	0.00 (0.0–0.0)	
API%			*p* = 0.00
mean ± SD	41.5 ± 18.0	27.4 ± 0.11.8	
median (Q1–Q3)	40.0 (30.0–54.0)	27.0 (20.0–35.0)	
SBI%			*p* = 0.01
mean ± SD	2.69 ± 8.45	4.75 ± 8.34	
median (Q1–Q3)	0.00 (0.00–0.00)	0.00 (0.00–11.0)	
Number of teeth to treat or extract	0.91 ± 1.76	0.51 ± 0.98	*p* = 0.33
Saliva cortisol			*p* = 0.13
concentration (ng/mL)	19.1 ± 3.5	19.6 ± 4.0	

Values are presented as X ± SD and median with the 1st quartile and 3rd quartile; *p* ≤ 0.05; Mann–Whitney U test***; N/A—not applicable. NS: not-statistical analysis.

**Table 2:** Characteristics of the parameters obtained from anamnesis.

Investigated Parameter	RRMS Group (n = 101)	Control Group (n = 51)	X^2^/*p*-Value
Swallowing problems	24.8%	1.96%	X^2^ = 12.4/*p* = 0.00
Pronunciation problems	32.7%	1.96%	X^2^ = 22.8/*p* = 0.00
Pain during mouth opening	3.96%	0.00%	X^2^ = 2.07/*p* = 0.15
Dry mouth feeling	42.6%	15.7%	X^2^ = 11.0/*p* = 0.00
Taste disturbances	10.9%	1.96%	X^2^ = 7.83/*p* = 0.02
Metallic taste feeling	12.8%	3.92%	X^2^ = 3.05/*p* = 0.08
Burning mouth sensations	1.98%	0.00%	X^2^ = 1.02/*p* = 0.312
Tooth pain	19.7%	25.5%	X^2^ = 1.11/*p* = 0.57
Gingival problems	25.7%	13.7%	X^2^ = 2.88/*p* = 0.08
RAS appearance	25.7%	17.6%	X^2^ = 1.25/*p* = 0.26
Labial herpes appearance	31.7%	35.3%	X^2^ = 0.20/*p* = 0.65
Self-assessment of oral hygiene			X^2^ = 5.05/*p* = 0.08
- Proper	25.7%	43.1%	
- Satisfactory	66.3%	53.0%	
- Bad	8.0%	3.9%	
Last visit to dental office			X^2^ = 5.42/*p* = 0.06
- Up to 6 months	44.6%	49.0%	
- During the last 12 months	32.7%	43.1%	
- Do not remember	22.7%	7.9%	
Toothbrushing frequency			X^2^ = 9.65/*p* = 0.02
- Twice daily	79.0%	92.1%	
- Once daily	18.0%	2.0%	
- After each meal	2.0%	5.9%	
- N/A	1.0%	0.0%	
Need help during tooth brushing	3.9%	0.0%	X^2^ = 2.61/*p* = 0.27
Bleeding during brushing	22.7%	23.5%	X^2^ = 0.51/*p* = 0.77
Using a solution for rinsing	44.5%	54.9%	X^2^ = 1.45/*p* = 0.22
Smoking habit	12.9%	11.8%	X^2^ = 0.03/*p* = 0.84
Prosthetic restorations			X^2^ = 4.84/*p* = 0.08
- Fixed	23.7%	25.5%	
- Movable	8.91%	0.0%	

**Table 3:** Correlations among the analyzed parameters in the RRMS group.

Correlation	Disease Duration (r; p)	Cortisol Concentration (r; p)	EDSS Scale (r; p)
Age (years)	0.47; 0.00	NS	0.58; 0.00
Disease duration (years)	-	NS	0.53; 0.00
Number of missing teeth	0.27; 0.00	NS	0.44; 0.00
Number of composite fillings	NS	NS	0.19; 0.06
Number of amalgam fillings	NS	NS	NS
Number of teeth treated or extracted	NS	NS	NS
API%	NS	NS	NS
SBI%	NS	NS	−0.23; 0.02
EDSS	0.53; 0.00	0.03; 0.73	-
Right-hand test (Nine Hole Pegs)	0.45; 0.00	NS	0.71; 0.00
Left-hand test (Nine Hole Pegs)	0.48; 0.00	NS	0.66; 0.00

**Legend:** NS: not-statistical analysis.

## Data Availability

The raw data supporting the conclusions of this article will be made available by the authors upon request.
